# The Diagnostic Accuracy of Electrical Impedance Spectroscopy-Assisted Colposcopy, HPV mRNA Test, and P16/Ki67 Immunostaining as CIN2+ Predictors in Greek Population

**DOI:** 10.3390/diagnostics14131379

**Published:** 2024-06-28

**Authors:** Nikolaos Tsampazis, Eleftherios Vavoulidis, Chrysoula Margioula-Siarkou, Marianthi Symeonidou, Stergios Intzes, Alexios Papanikolaou, Konstantinos Dinas, Angelos Daniilidis

**Affiliations:** 1 2nd Department of Obstetrics & Gynecology, Medical Faculty, Aristotle University of Thessaloniki, Hippokration General Hospital, 56429 Thessaloniki, Greece; tsabazis@yahoo.com (N.T.); margioulasiarkouc@gmail.com (C.M.-S.); marianthi.symeonidou@hotmail.com (M.S.); instergios@hotmail.com (S.I.); anpapani@auth.gr (A.P.); konstantinosdinas@hotmail.com (K.D.); 2 1st Department of Obstetrics & Gynecology, Medical Faculty, Aristotle University of Thessaloniki, Papageorgiou General Hospital, 54124 Thessaloniki, Greece; angedan@hotmail.com

**Keywords:** electrical impedance spectroscopy, CIN2 lesions, Greek patients, HPV mRNA test, ZedScan, P16/Ki67 immunostaining

## Abstract

Objective: To evaluate the diagnostic accuracy of Electrical Impedance Spectroscopy (EIS)-assisted colposcopy in detecting CIN2+ Greek women towards standalone colposcopy, HPV mRNA testing, and p16/Ki67 immunostaining. Methods: We conducted a cross-sectional observational study at the Cervical Pathology Clinic of the 2nd Obstetrics-Gynecology University Department of Hippokration Hospital Thessaloniki involving 316 patients from January 2022 to August 2023. All participants provided liquid-based cervical samples for cytology, HPV mRNA testing, and p16/Ki67 immunostaining. Main Outcome Measures: Subsequently, participants underwent both standalone colposcopy and EIS/ZedScan-assisted colposcopy, followed by cervical punch biopsies. Results: The incorporation of EIS significantly enhanced the sensitivity of colposcopy, increasing it from 54.17% to 100%, equivalent to that of HPV mRNA testing and p16/Ki67 immunostaining, while achieving a high specificity (95.45%). The specificities observed with EIS/ZedScan-assisted and standalone colposcopy were notably superior to those of HPV-related biomarkers (HPV mRNA test and p16/Ki67 immunostaining). When compared to standalone colposcopy, HPV mRNA testing, and p16/Ki67 immunostaining, EIS/ZedScan-assisted colposcopy demonstrated the most favorable combination of Positive and Negative Predictive Values, at 90.57% and 100%, respectively. The inclusion of EIS/ZedScan in colposcopy led to the detection of 44 additional cases of true CIN2+ (100% of the total CIN2+ confirmed histologically) that were missed by standalone colposcopy. This discovery suggests a 45.83% increase in the detection of CIN2+ cases. Conclusions: The integration of EIS with colposcopy has demonstrated effectiveness in detecting cervical lesions, resulting in a significant detection increase of CIN2+ cases while offering optimal levels of sensitivity, specificity, and predictive values for CIN2+ detection.

## 1. Introduction

Cervical cancer (CxCa) is a significant global health problem affecting women, ranking as the fourth most common cancer after breast, colorectal, and lung cancers [1,2]. This remains true despite the considerable impact of incorporating HPV testing alongside the pap test as a co-testing approach in recent years, which has significantly improved the acceptance of CxCa screening [3,4,5].

Human papillomavirus (HPV), particularly its high-risk subtypes (hr-HPVs), has been identified as the primary causative agent of cervical cancer (CxCa), owing to its notable proclivity for neoplastic growth [6]. Generally, more than half of sexually active women contract HPV infection at some point in their lives [7]. Although most HPV infections are transient and lack cytological abnormalities, long-term persistent infections caused by hr-HPVs, including HPV-16, HPV-18, and HPV-45, are strongly associated with the development of high-grade squamous intraepithelial lesions (HSIL/CIN) and CxCa [8]. Currently, the market offers a diverse array of methodologies for the identification and characterization of DNA and mRNA molecules from hr-HPVs [9,10].

When it comes to predicting CIN2+, molecular hr-HPV screening has shown superior accuracy in comparison to cytology alone, as evidenced by several studies [11,12,13]. Despite the substantial reduction in the burden of CxCa through co-testing initiatives [12], colposcopy and biopsies remain effective diagnostic methods for identifying CINs and CxCa [14,15]. Nonetheless, the assessment of colposcopy’s performance is complex due to the need to excise the transformation zone (TZ) to determine disease status, coupled with a wide range of specificity (29–94%), primarily attributed to the subjectivity and expertise of the colposcopist [16,17,18]. Several studies have suggested that the detection of CIN2+ may not always be straightforward, particularly in cases of small or limited lesions. In fact, research findings indicate that approximately 45% of CIN2+ cases are identified through random punch biopsies rather than colposcopy-guided biopsies [17,19,20]. Given the relatively low sensitivity and specificity of these existing methods, there arises a need for more precise diagnostic techniques in the context of precancerous lesions detection.

The utilization of Electrical Impedance Spectroscopy (EIS) on epithelial cells holds promise as a viable approach [21]. EIS is an electrokinetics-based method that offers a non-invasive means of detecting abnormal cells, monitoring their behavior, and studying their various attributes. This technique operates by passing an ionic electrical current through the extracellular space around cells and, at higher frequencies, across the cell membranes into the intracellular space [22,23]. Malignant transformation leads to structural changes of the cells, affecting their electrical conductivity and, consequently, their electrical impedance. This enables the real-time measurement of their dynamic behavior [24,25]. Empirical research has widely applied EIS in different anatomical locations, such as the breast [26], skin [27], and lung tissue [28]. Various studies have proposed the potential use of EIS in Gynecology, suggesting that EIS can serve as a precise and expedient method for detecting abnormal cellular changes in the cervical epithelium [29,30,31,32].

In addition to changes in cell stratification and differentiation and an increased nuclear–cytoplasmic ratio, the growth of cervical intraepithelial neoplasia (CIN) results in a significant expansion of the extracellular space, up to six times the size of normal tissue. This expansion leads to a reduction in impedance at low frequencies within abnormal tissues. Notably, high-grade CIN exhibits lower impedance compared to normal epithelium [30,31]. The growing interest in EIS within the field of Gynecology has led to the development of ZedScan, a portable device designed to assess EIS on the cervical epithelium, aiding in the differentiation between normal and pathological cells. Preliminary investigations have suggested that the ZedScan prototype can enhance the performance of colposcopy in detecting CIN2+ [29,33]. In 2013, ZedScan was integrated into regular clinical practice with the aim of providing colposcopists with more information, thereby facilitating more effective patient treatment [34].

In a previous study, we conducted a comparative analysis of the diagnostic parameters of EIS/ZedScan in the detection of HSILs/CIN2+ among Greek women, comparing them to colposcopy alone and HPV mRNA testing. The initial findings indicated that ZedScan significantly improved the detection of CIN2+ when compared to colposcopy performed in isolation [35]. The objective of this study is to assess the accuracy of EIS/ZedScan in detecting CIN2+ in a broader population sample while comparing its performance to standalone colposcopy, HPV mRNA testing, and an additional HPV-related biomarker, p16/Ki67 immunostaining, which has recently been recommended for inclusion in CxCa screening strategies [36].

## 2. Materials and Methods

The present study is a cross-sectional, single-center, observational investigation that examined a total of 316 females who were patients at the Colposcopy and Cervical Pathology Clinic of the 2nd Obstetrics-Gynecology Department, Aristotle University of Thessaloniki, located at Hippokration General Hospital (Thessaloniki, Greece). The patient recruitment and data collection period for this study spanned from January 2022 to August 2023. The study group consisted of individuals who were referred to colposcopy, mostly due to an abnormal pap test. All patients gave written consent to participate in this study after being properly informed by the clinician.

The study recruited female participants who met specific criteria: (a) being at least 18 years old; (b) having no clinical history of cervical cancer or precancerous lesions; (c) not having undergone any cervical treatment; (d) not being pregnant; and (e) having undergone an adequate colposcopic examination with a TZ of type 1 or 2, as defined by the Hellenic Society for Colposcopy & Cervical Pathology. The methodologies/techniques used in research involving human subjects adhered to the ethical principles outlined in the 1964 Helsinki Declaration and its subsequent revisions, or equivalent ethical standards. The study protocol has received approval from the Bioethics Research Committee on human research of Thessaloniki Medical School at Aristotle University of Thessaloniki (protocol number 44/14-12-2022, approval date 14 December 2022).

Cervical exfoliated cell samples, which were collected using a sample brush from the cervical surface, were utilized for cytology, HPV mRNA test, and P16/Ki67 immunostaining. These samples were obtained prior to the commencement of colposcopy, EIS, and punch biopsy procedures, all of which were performed by experienced colposcopists.

All participants had colposcopy utilizing a Binocular colposcope D100 3000 equipped with an AVC 561 Digital Color CCD Camera and Colpo ITPro software v.2.0 for picture collecting and processing (Dormed Hellas, Thessaloniki, Greece), as per the established local protocol. The process of video recording was conducted on two separate occasions, namely prior to and after the administration of a 3% acetic acid solution.

Subsequently, EIS analysis was conducted using ZedScan (Zilico Limited, Manchester, UK), in accordance with the guidelines provided by the manufacturer. ZedScan is a compact handheld instrument equipped with a sensor located at its tip, designed for single-use purposes. EIS was conducted using a set of four electrodes, whereby the device’s tip was positioned onto the cervical epithelium. Twelve measurements were conducted on the TZ for each patient, with all data being visually presented on the device’s screen and subsequently saved for future reference. The device employed a grading system consisting of three colors to identify regions with the greatest likelihood of HSIL occurrence. The highest probability areas were represented by red, while amber indicated a lower probability and green indicated the lowest probability. This system aided in the selection of potential areas for biopsy sampling. The utilization of single-point mode was employed to identify the specific regions for biopsy specimens in instances when a red signal was present. ZedScan was considered to yield positive results for CIN when an amber or red hue was observed, whereas negative results were obtained when a green color was observed.

When the colposcopy results were negative and only ZedScan-directed biopsies found CIN2+ regions, this was regarded as an augmentation in the detection rate of CIN2+ by ZedScan-assisted colposcopy compared to standalone colposcopy. Any woman who had negative results for both colposcopy and ZedScan was classified as a negative case for CIN2+. Although biopsy sampling is generally not recommended in these cases, it was performed nevertheless due to the suspicious cytological referral (ASCUS, LSIL, ASC-H). The purpose of these biopsies was to utilize the histology reports as the endpoint for comparing the diagnostic accuracy of the two colposcopy modes and the two molecular biomarkers. The colposcopy procedure was concluded by applying Lugol’s solution to the surface of the cervix to detect any potential abnormalities without causing stains. Biopsies were performed in accordance with the colposcopic impression and/or ZedScan results, and afterwards submitted to the Pathology Lab of the Hospital for histological evaluation.

The cytological specimen was conserved using PreservCyt/ThinPrep solution (Cytyc Corporation, Marlborough, MA, USA). The utilization of liquid-based cytology (LBC) technology with a Thin Prep 2000 Processor (Cytyc Corporation) was employed in accordance with the provided instructions to prepare thin-layer slides. These slides were subsequently stained using the Pap method and evaluated by experienced cytologists at the Hospital. The assessment was conducted based on the criteria outlined in the third edition of the Bethesda System for Reporting Cervical Cytology [37].

An additional cytology slide was generated for p16/Ki67 immunostaining from the remaining LBC material using the Thin Prep 2000 Processor and positively charged cytology slides (Hologic, Marlborough, MA, USA). The p16/Ki67 immunostaining was performed using a CINtec Plus Kit (Roche laboratories AG, Mannheim, Germany) in accordance with the manufacturer’s instructions on the BenchMark GX IHC ISH System (Ventana Medical Systems Inc., Marana, AZ, USA). The cervical cytology slides were analyzed by the cytopathologists of the Hospital to assess the staining efficacy of two markers. A case was deemed positive for p16/Ki67 immunostaining if there was at least one cervical epithelial cell that exhibited both a brown cytoplasmic stain (indicative of p16) and a red nuclear stain (indicative of Ki67). Slides without brown cytoplasmic staining and/or a red nuclear stain were classified as negative.

The Laboratory of Gynecologic Oncology of the Clinic conducted all the molecular analyses. In detail, a volume of 1 milliliter was manually transferred from each Thin Prep sample to an Aptima Specimen Transfer Tube. Subsequently, the samples were placed into the Panther system (Hologic, Marlborough, MA, USA). The mRNA Aptima assay (Hologic, Marlborough, MA, USA) was employed in accordance with the guidelines provided by the manufacturer considering any signal-to-cut-off value >1.0 as a positive test.

The data analysis was conducted using SPSS software (Version 24). Statistical analyses were employed as suitable, encompassing descriptive statistics, Chi-square test, Fisher’s exact test, using a two-tailed approach. Chi-square tests were employed to assess the disparities in percentages across the groups. Histological confirmation of CIN2+ has been established as the definitive gold standard for cervical disease. The variables were quantified to conduct Spearman’s Correlation. The variable of Cytology was categorized into five ranks: (a) NILM, (b) ASC-US, (c) LSIL, (d) ASC-H and HSIL, and (e) SCC. On the other hand, the variables Colposcopy and Histology were classified into three ranks: (a) Negative, (b) LSIL (CIN1), and (c) HSIL (CIN2, CIN3, or SCC). HPV mRNA test and p16/Ki67 immunostaining were classified into two ranks: (a) Negative and (b) Positive. Diagnostic sensitivity and specificity were determined for the detection of CIN2+ using 2 × 2 tables, and the findings are shown together with 95% confidence intervals for all tests. The Receiver Operating Characteristic (ROC) curve was employed to assess and evaluate the diagnostic accuracy of various assays, with the objective of determining the optimal diagnostic threshold for both colposcopy modes and HPV-related biomarkers. A *p*-value < 0.05 was used to indicate statistical significance for all analyses.

## 3. Results

Demographics: Most participants in the study were of Caucasian ethnicity, accounting for 91.2% of the sample. Their ages ranged from 19 to 72 years, with a mean age of 32.1 years. Most of the participants (53.8%) reported being frequent smokers, while a significant proportion (58.2%) stated that they were not taking any preventive measures. The study found that individuals engaged in their first sexual intercourse at ages ranging from 13 to 34 years, with a mean age of 17.1 years. Among the sample of 316 participants, 194 individuals (61.4%) reported having been sexually involved with at least three different partners and having given birth to at least two children. Regarding HPV vaccination, approximately 30.4% (*n* = 96) of the participants received immunization. Table 1 provides an in-depth representation of the demographic characteristics.

Cytological findings: A significant proportion of the participants (*n* = 176, 55.7%) were referred for further evaluation based on a LSIL pap test. The remaining participants were evenly distributed between those referred for high-grade abnormalities (HSIL or ASC-H) (22.2%) and those for minor abnormalities (ASC-US) (21.5%), while two cases of SCC were identified. The pap tests conducted by the cytologists at our facility exhibited a lack of complete concordance with the referral findings. The cytology team provided a comprehensive diagnosis, identifying 162 cases of LSIL, 83 cases of HSIL or ASC-H, 48 cases of ASCUS, 3 cases of SCC and 20 cases categorized as negative for intraepithelial lesions. Table 2 displays the cytological findings.

Colposcopical findings: All patients received a thorough colposcopy examination, which was afterwards followed by an evaluation using EIS/ZedScan (Table 3). The colposcopy alone identified 49 individuals, accounting for 15.5% of the total, who had CIN2+ lesions. Additionally, there were 2 cases of SCC and 206 CIN1/LSIL cases, making up 65.2% of CIN1/LSIL. The utilization of EIS/ZedScan resulted in a notable rise in the incidence of CIN2+ cases, increasing from 49 to 103 (representing a 17.1% increase). Conversely, there was a decrease in the number of patients diagnosed with CIN1/LSIL colposcopy, declining from 206 to 166 (a reduction of 12.7%). Additionally, the deployment of EIS/ZedScan led to a decrease in the number of patients with negative colposcopy, declining from 58 to 44 (a decrease of 4.4%). There was no observed alteration in the count of SCC following the implementation of ZedScan (*n* = 3, 0.9%).

HPV mRNA test results: The findings of the HPV mRNA test indicated that most women (*n* = 212, 67.1%) tested positive for HPV mRNA molecules. Out of the total of 212 women who tested positive, approximately half of them (*n* = 116, 54.7%) were found to be positive for HPV-16. Additionally, 48 women (22.7%) tested positive for HPV-18/45, while 10 women were positive for both HPV-16 and HPV-18/45. In the remaining 38 positive cases, HPV mRNA from high-risk HPV types other than HPV-16/18/45 was detected. No statistically significant associations were seen for any of the high-risk HPV genotypes in terms of the two colposcopy modes. In a comprehensive analysis, it was observed that there was no significant association between standalone colposcopy and any specific HPV genotype. The *p*-values obtained for HPV-16, HPV-18/45, co-infection of HPV-16 and 18/45, and other hr-HPVs were 0.129, 0.213, 0.099, and 0.347, respectively. The *p*-values associated with EIS/ZedScan-assisted colposcopy were 0.061, 0.189, 0.094, and 0.298, in the same order. A summary of the HPV mRNA test findings can be seen in Table 4.

p16/Ki67 immunostaining: The p16/Ki67 immunostaining revealed that 33.7% (*n* = 138) of the sample exhibited a positive dual stain diagnostic for p16/Ki67, indicating the presence of advanced HPV infection. Table 4 shows the findings for all p16/Ki67 diagnoses.

Histological findings: Punch biopsies were taken from all 316 patients, as indicated in Table 3. The histological findings were employed as the gold standard for evaluating the performance of the two colposcopy modes as well as the HPV-related biomarkers. In a comprehensive analysis, it was shown that most cases were diagnosed as CIN1/LSIL (*n* = 178, 56.4%). Additionally, 93 cases (29.4%) were categorized as CIN2 or above. In conclusion, a total of 42 histological specimens were determined to be negative for cervical intraepithelial lesions, accounting for 13.3% of the sample, and three cases (0.9%) were diagnosed as SCC.

Statistical analysis: Most characteristics examined did not exhibit statistical significance in relation to either the two colposcopy modes or HPV-related biomarkers, except for the utilization of prophylactics and the age at which individuals first engaged in sexual intercourse. The results presented in Table 5 indicate a statistically significant correlation between age at first sexual intercourse and the use of prophylactics with colposcopy alone (*p* = 0.041 and 0.020, respectively), EIS/ZedScan-assisted colposcopy (*p* = 0.021 and 0.035, respectively), HPV mRNA test (*p* = 0.048 and 0.040, respectively), and p16/Ki67 immunostaining (*p* = 0.032 and 0.029, respectively). Regarding the other factors, statistical analysis revealed no significant associations between age (*p* = 0.324), number of children (*p* = 0.087), number of lifetime sexual partners (*p* = 0.065), smoking (*p* = 0.073), and HPV vaccination (*p* = 0.098) with respect to colposcopy alone. In the case of ZedScan, there was no observed statistical significance for age (*p* = 0.235), number of children (*p* = 0.092), number of lifetime sexual partners (*p* = 0.074), smoking (*p* = 0.065), and HPV vaccination (*p* = 0.076). The corresponding *p*-values for the HPV mRNA test were 0.127, 0.109, 0.087, 0.061, and 0.192, whereas the *p*-values for the p16/Ki67 immunostaining were 0.314, 0.109, 0.089, 0.081, and 0.087, as shown in Table 5.

Given the ordinal nature of the variables being analyzed, we employed Spearman’s ρ (rho) non-parametric correlation coefficient. A value near −1 indicates a strong negative correlation, while a value near +1 indicates a strong positive correlation. The results presented in Table 5 indicate that there are statistically significant variables associated with diagnostic procedures/tests that exhibit a strong positive correlation. Specifically, these variables include the utilization of prophylactics, as evidenced by rho values ranging from 0.798 to 0.892, and the age at which individuals first engage in sexual intercourse, with rho values ranging from 0.749 to 0.857. These findings suggest that the absence of prophylactic use as well as an earlier initiation of sexual activity are factors that may increase the likelihood of developing CIN2+. The correlation coefficients of the remaining variables ranged from 0.500 to 0.700, indicating a moderate level of correlation, or below 0.500, indicating a low level of correlation.

By integrating the outcomes of colposcopy, EIS/ZedScan, HPV-related biomarkers, and histological analysis, we computed various diagnostic parameters, including sensitivity, specificity, positive predictive value (PPV), and negative predictive value (NPV), for all four tests (Table 6). The endpoint for these calculations was the histologically confirmed diagnosis of CIN2+, as indicated in Table 3. The implementation of EIS significantly improved the sensitivity of colposcopy, increasing it from 54.17% to 100%, which is equivalent to the corresponding values of the HPV mRNA test and p16/Ki67 immunostaining. Furthermore, EIS maintained a high specificity, with colposcopy achieving 100% and EIS/ZedScan achieving 95.45%. These specificity values were notably better than those seen for HPV-related biomarkers, which had specificity values of 47.27% (HPV mRNA test) and 80.91% (p16/Ki67 immunostaining). In comparison to colposcopy alone, HPV mRNA test, and p16/Ki67 immunostaining, EIS-assisted colposcopy demonstrated the most favorable combination of PPV and NPV, with values of 90.57% and 100%, respectively. Colposcopy alone exhibited PPV and NPV values of 100% and 83.33%, while HPV mRNA test and p16/Ki67 immunostaining yielded PPV and NPV values of 45.28% and 100%, and 69.57% and 100%, respectively. All the corresponding *p*-values were equal to 0.00 < 0.05.

## 4. Discussion

The introduction of EIS/ZedScan significantly improved the sensitivity of colposcopy from 54.17% to 100%, which is comparable to that of HPV-related biomarkers. Additionally, the adoption of EIS/ZedScan maintained a high specificity value (95.45%), which is considerably higher than the values of HPV mRNA testing (47.27%) and p16/Ki67 immunostaining (80.91%). In our previous study, the introduction of EIS was found to significantly improve sensitivity of colposcopy, increasing it from 80.65% to 100% [35]. The elevated standard deviation observed in the study can primarily be attributed to the limited sample size of only 86 women, nearly four times smaller than the sample size in the current investigation. Furthermore, the National Health Service provides cervical screening services to women aged 25–65. However, women < 25 years are only sent to colposcopy if they exhibit symptoms or indicators of CxCa. The study population in our research aligns with this practice, as indicated by a mean age at referral of 32.1 years (19 to 72 years).

It is worth noting that EIS-assisted colposcopy demonstrated the most favorable combination of PPV and NPV, at 90.57% and 100%, respectively. In comparison, standalone colposcopy exhibited a PPV of 100% and an NPV of 83.33%, while HPV mRNA testing showed a PPV of 45.28% and an NPV of 100%. Lastly, p16/Ki67 immunostaining testing yielded a PPV of 69.57% and an NPV of 100%. Muszyński et al. conducted an evaluation of ZedScan in the context of colposcopical examination among 91 women exhibiting abnormal cervical cytology in France [38]. The study reported sensitivity of 93.1% and NPV of 91.3%. These findings align with our research, as well as with the results of a study conducted in Poland [39]. That study of 118 women concluded that colposcopy yielded sensitivity of 96.30% and NPV of 97.30% [39]. Additionally, the reported specificity of 95.45% for EIS/ZedScan-assisted colposcopy is consistent with the findings of Tidy et al., who observed a specificity of 95.4% in a sample of involving 474 British women [34]. This is also in line with our previous study of 86 women, where the sensitivity was determined to be 94.74% [35].

There is a consensus among experts that high-grade disease associated with HPV16 exhibits a more accelerated progression and is more prevalent among younger women towards diseases caused by other hr-HPVs. Consequently, this facilitates its earlier and more readily detectable identification during colposcopy procedures. However, this study demonstrates that CIN2+ detection rates remained consistently high irrespective of the hr-HPV genotype present. These findings suggest that ZedScan’s performance is not influenced by the presence of hrHPV. The *p*-values corresponding to EIS/ZedScan-assisted colposcopy were 0.061 for HPV-16, 0.189 for HPV-18/45, 0.094 for the co-infection of HPV-16 and 18/45, and 0.298 for other high-risk HPV types. These results did not demonstrate statistical significance, which aligns with findings from previous studies [40,41]. The findings of several studies [40,42] indicate that HSILs with HPV-16 exhibit a tendency for accelerated development and are more frequently observed in younger women, such as those included in this study. This characteristic makes it feasible to identify potential lesions at an earlier stage and with greater ease during colposcopy. Therefore, the findings provide an explanation for the prevalence of HPV-16, which was diagnosed in 126 out of 316 women, accounting for most of the patients (59.4%). The utilization of colposcopy has been found to be more effective in detecting high-grade disease associated with HPV16 in comparison to non-HPV16 disease. However, it is important to consider that this discrepancy may be influenced by factors such as the age distribution and disease prevalence within the specific group under investigation. According to the study, the application of acetic acid did not yield any statistically significant alterations in the impedance spectra data as reported by other studies [43].

The objective of implementing a well-functioning CxCa screening program is to identify + CIN2 promptly and conveniently as well as any abnormalities with colposcopy [38]. This approach facilitates timely intervention and ultimately serves to mitigate the progression of cervical cancer [44]. The significance of a high sensitivity for colposcopy depends on its ability to accurately detect all cases of CIN2+. With the enhanced accuracy of HPV testing compared to cytology alone, an effective program would have the capability to identify a greater number of women with CIN2+ via colposcopy at an earlier stage, prior to the detection of the disease [45,46,47]. This highlights the necessity for a diagnostic examination that is not solely reliant on visual assessment of acetowhite changes [48].

The utilization of EIS in conjunction with colposcopies has emerged as a promising and creative approach for the accurate diagnosis of cervical lesions [18,49,50]. The ZedScan device utilizes EIS to distinguish between normal and pathological cervical cell structures. This differentiation is achieved by analyzing differences in spectra profiles, which are indicative of cellular alterations associated with cervical intraepithelial neoplasia [21,22,23]. It is important to acknowledge that ZedScan demonstrates a favorable safety profile, as it is both painless and devoid of any adverse events [29,30,31,32]. In addition, the real-time acquisition of results by ZedScan plays a key role in reducing the emotional strain related to the turnaround time of diagnostic data [49]. In conclusion, it is important to highlight that the cost of a single-use EIS sensor for each colposcopy is approximately GBP 30. Furthermore, an economic analysis conducted by Peron et al. reported that the incremental cost-effectiveness ratio for ZedScan ranged from GBP 272 to GBP 4922 per quality-adjusted life-year [51].

Our findings indicate that a total of 96 women with histologically confirmed CIN2+ were identified using EIS/ZedScan-assisted colposcopy, with additionally 10 more women who were histologically diagnosed with CIN1. It is noteworthy that only 52 women, accounting for 54.17% of the total CIN2+ cases, were diagnosed solely through standalone colposcopy. The implementation of EIS led to the identification of 44 additional cases of true CIN2+ (representing 100% of the total CIN2+ cases histologically confirmed) that were not detected by standalone colposcopy, which classified these 44 cases either as CIN1 (40/44) or Negative (4/44)). This suggests a 45.83% increase in the detection of CIN2+ cases, which is consistent with the 47.3% increase reported by Muszyński et al. [38]. A recent study conducted by Tidy et al. revealed that ZedScan successfully identified an additional 269 cases of CIN2+ patients, or 24% of the total cases [49]. The apparent disagreement in our findings could potentially be explained by the significant difference in the sample sizes used in the two studies (5257 compared to 316 in our study).

One notable aspect of this study is its novelty, as it appears to be the first known attempt to evaluate and assess the diagnostic effectiveness of implementing EIS in colposcopy compared to standalone colposcopy and two commonly utilized HPV-related biomarkers, HPV mRNA testing and p16/Ki67 immunostaining, specifically in terms of detecting CIN2+. However, it is important to acknowledge that novel technologies also possess certain constraints. The proficiency of the colposcopist remains still a crucial determinant not only in accurately assessing the cervix, but also in identifying and diagnosing conditions affecting the vaginal walls, vulva, and anus, as well as cases of type 3 TZs, which are presently not encompassed by emerging technologies. So, currently, the complete replacement of skilled colposcopic practice remains imperative.

## 5. Conclusions

The utilization of colposcopies with EIS has exhibited efficacy in the detection of cervical lesions, resulting in a notable augmentation in the identification of CIN2+ cases that would have otherwise been missed by colposcopy only. The diagnostic threshold of EIS/ZedScan appears to exhibit optimal levels of sensitivity, specificity, and predictive values for the identification of CIN2+ when compared to standalone colposcopy, HPV mRNA testing, and p16/Ki67 immunostaining, emphasizing its value as a diagnostic tool for clinicians in their endeavor to combat cervical cancer.

## Figures and Tables

**Table 1:** Sample demographics.

	Mean (Minimum–Maximum)
Age (years)	32.1 (19–72)
Age at first sexual intercourse (years)	17.1 (13–34)
	***n* (N%)**
Number of children	
0	74 (23.4%)
1	48 (15.2%)
2	98 (31.0%)
3	72 (22.8%)
4	24 (7.6%)
Number of lifetime sexual partners	
1	24 (7.6%)
2	98 (31.0%)
3	62 (19.6%)
4	88 (27.9%)
>4	44 (13.9%)
Smoking	
Yes	170 (53.8%)
No	146 (46.2%)
Use of prophylactics	
Yes	132 (41.8%)
No	184 (58.2%)
HPV vaccination	
Yes	96 (30.4%)
No	220 (69.6%)

**Table 2:** Cytological results.

Referral Cytology	*n* (N%)	Cytology	*n* (N%)
NILM	0 (0.0%)	NILM	20 (6.3%)
ASC-US	68 (21.5%)	ASC-US	48 (15.2%)
ASC-H	22 (7.0%)	ASC-H	16 (5.1%)
LSIL	176 (55.7%)	LSIL	162 (51.3%)
HSIL	48 (15.2%)	HSIL	67 (21.2%)
SCC	2 (0.6%)	SCC	3 (0.9%)
Total	316 (100%)	Total	316 (100%)

**Table 3:** Colposcopical and EIS/ZedScan results.

Colposcopy Alone	*n* (N%)	EIS/ZedScan-Assisted Colposcopy	*n* (N%)	Histology	*n* (N%)
NEGATIVE	58 (18.4%)	NEGATIVE	44 (14.0%)	NEGATIVE	42 (13.3%)
CIN1/LSIL	206 (65.2%)	CIN1/LSIL	166 (52.5%)	CIN1/LSIL	178 (56.4%)
CIN2/CIN3/HSIL	49 (15.5%)	CIN2/CIN3/HSIL	103 (32.6%)	CIN2/CIN3/HSIL	93 (29.4%)
SCC	3 (0.9%)	SCC	3 (0.9%)	SCC	3 (0.9%)
Total	316 (100%)	Total	316 (100%)	Total	316 (100%)

**Table 4:** HPV-related biomarkers results.

HPV mRNA Test	*n* (N%)	p16/Ki67 Immunocytochemistry Results	*n* (N%)		
NEGATIVE	104 (32.9%)	NEGATIVE	178 (56.3%)		
POSITIVE	212 (67.1%)	POSITIVE	138 (33.7%)		
Total	316 (100%)	Total	316 (100%)		
**Women with positive HPV mRNA**		**CIN2+ by colposcopy** **alone**	***p*-value**	**CIN2+ by EIS/ZedScan-assisted colposcopy**	***p*-value**
HPV-16 (+)	116 (54.7%)	37 (71.2%)	0.129	84 (79.2%)	0.061
HPV-18/45 (+)	48 (22.7%)	3 (5.7%)	0.213	6 (5.7%)	0.189
HPV-16 (+) and HPV-18/45 (+)	10 (4.7%)	8 (15.5%)	0.099	10 (9.4%)	0.094
other hr-HPV (+)	38 (17.9%)	4 (7.6%)	0.347	6 (5.7%)	0.298
Total	212 (100%)	52 (100%)		106 (100%)	

**Table 5:** Spearman’s rho statistical correlations of colposcopy modes and HPV-related biomarkers correlated with each variable.

	Colposcopy Alone		EIS/ZedScan-Assisted Colposcopy		HPV mRNA Test		p16/Ki67 Immunostaining	
Parameter	*p*	Spearman’s Rho ρ	*p*	Spearman’s Rho ρ	*p*	Spearman’s Rho ρ	*p*	Spearman’s Rho ρ
Age	0.324	0.268	0.235	0.125	0.127	0.388	0.314	0.297
Age at first sexual intercourse	**0.041**	**0.802**	**0.021**	**0.749**	**0.048**	**0.857**	**0.032**	**0.796**
Number of children	0.087	0.397	0.092	0.371	0.109	0.297	0.109	0.274
Number of lifetime sexual partners	0.065	0.448	0.074	0.658	0.087	0.602	0.089	0.591
Smoking	0.073	0.287	0.065	0.357	0.061	0.279	0.081	0.263
Use of prophylactics	**0.020**	**0.845**	**0.035**	**0.892**	**0.040**	**0.798**	**0.029**	**0.841**
HPV vaccination	0.098	0.159	0.076	0.186	0.192	0.349	0.087	0.293

**Table 6:** Diagnostic parameters for colposcopy, ZedScan, and HPV mRNA test using as threshold CIN2+ histologically confirmed.

	Colposcopy Alone	95% CI	EIS/ZedScan-Assisted Colposcopy	95% CI	HPV mRNA Test	95% CI	p16/Ki67 Immunostaining	95% CI
Sensitivity	54.17%	43.69–64.38%	100.00%	96.23–100.00%	100.00%	96.23–100.00%	100.00%	96.23–100.00%
Specificity	100.00%	98.34–100.00%	95.45%	91.80–97.80%	47.27%	40.52–54.10%	80.91%	75.08–85.88%
PPV	100.00%	93.15–100.00%	90.57%	83.97–94.62%	45.28%	42.20–48.40%	69.57%	63.52–75.00%
NPV	83.33%	80.09–89.70%	100.00%	98.26–100.00%	100.00%	96.52–100.00%	100.00%	97.95–100.00%

CI: Confidence Interval; PPV: Positive Predictive Value; NPV: Negative Predictive Value.

## Data Availability

The raw data supporting the conclusions of this article will be made available by the authors on request.
